# NeuroCave: A web-based immersive visualization platform for exploring connectome datasets

**DOI:** 10.1162/netn_a_00044

**Published:** 2018-09-01

**Authors:** Johnson J. G. Keiriz, Liang Zhan, Olusola Ajilore, Alex D. Leow, Angus G. Forbes

**Affiliations:** Department of Computer Science, University of Illinois at Chicago, Chicago, IL, USA; Collaborative Neuroimaging Environment for Connectomics, University of Illinois Chicago, Chicago, IL, USA; Department of Engineering and Technology, University of Wisconsin–Stout Menomonie, WI, USA; Collaborative Neuroimaging Environment for Connectomics, University of Illinois Chicago, Chicago, IL, USA; Department of Psychiatry, University of Illinois at Chicago, Chicago, IL, USA; Collaborative Neuroimaging Environment for Connectomics, University of Illinois Chicago, Chicago, IL, USA; Department of Computer Science, University of Illinois at Chicago, Chicago, IL, USA; Department of Psychiatry, University of Illinois at Chicago, Chicago, IL, USA; Collaborative Neuroimaging Environment for Connectomics, University of Illinois Chicago, Chicago, IL, USA; Department of Computer Science, University of Illinois at Chicago, Chicago, IL, USA; Collaborative Neuroimaging Environment for Connectomics, University of Illinois Chicago, Chicago, IL, USA; Computational Media Department, University of California, Santa Cruz, Santa Cruz, CA, USA

**Keywords:** Connectome visualization, Immersive analytics, Intrinsic geometry, Network analysis

## Abstract

We introduce NeuroCave, a novel immersive visualization system that facilitates the visual inspection of structural and functional connectome datasets. The representation of the human connectome as a graph enables neuroscientists to apply network-theoretic approaches in order to explore its complex characteristics. With NeuroCave, brain researchers can interact with the connectome—either in a standard desktop environment or while wearing portable virtual reality headsets (such as Oculus Rift, Samsung Gear, or Google Daydream VR platforms)—in any coordinate system or topological space, as well as cluster brain regions into different modules on-demand. Furthermore, a default side-by-side layout enables simultaneous, synchronized manipulation in 3D, utilizing modern GPU hardware architecture, and facilitates comparison tasks across different subjects or diagnostic groups or longitudinally within the same subject. Visual clutter is mitigated using a state-of-the-art edge bundling technique and through an interactive layout strategy, while modular structure is optimally positioned in 3D exploiting mathematical properties of platonic solids. NeuroCave provides new functionality to support a range of analysis tasks not available in other visualization software platforms.

## Author Summary

NeuroCave is a web-based software application that facilitates the exploration of and comparison between connectome datasets in virtual reality environments. This article describes the visualization features provided by NeuroCave, enabling clinical neuroscientists to investigate connectome datasets in a flexible manner, to detect patterns within connectome data, and to generate hypotheses about these observations. NeuroCave is open source software freely available via our GitHub code repository at https://github.com/CreativeCodingLab/NeuroCave.

## INTRODUCTION

Modern, noninvasive neuroimaging techniques provide a means with which to understand structural and functional brain networks, or connectomes (Sporns, Tononi, & Kötter, 2005). Diffusion MRI–derived white matter interconnectivity between different brain regions yields the structural connectome, and BOLD signal correlations generate the functional connectome. Mathematically, a connectome can be modeled as a graph by representing the different brain regions as nodes. Such models enable neuroscientists to apply network-theoretic methods and metrics, revealing important properties of the brain, such as small-worldness (Achard, Salvador, Whitcher, Suckling, & Bullmore, 2006; Salvador et al., 2005), clustering and modularity (Meunier, Lambiotte, & Bullmore, 2010), and rich-club configuration (Van Den Heuvel & Sporns, 2011), and embeddedness (Ye, Zhan, et al., 2015) among others.

**Figure 1.**
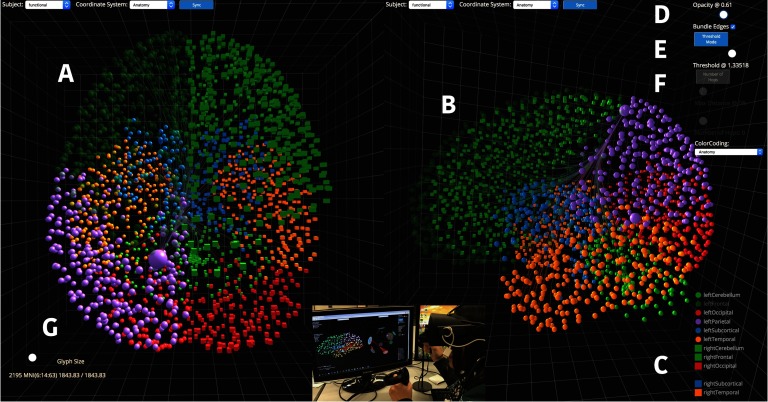
The user interface of NeuroCave, presenting multiple views to investigate connectome data. A researcher can compare different datasets or the same dataset from different perspectives. Here, (A) shows a high-resolution functional connectome, and (B) shows the same connectome from a different orientation. When viewing multiple copies of the same dataset, actions can be synchronized so that interacting with one connectome updates the other. Users can choose an atlas to label brain regions. (C) shows the color/glyph atlas, and a user can toggle on or off specific brain regions or classifications by clicking next to items in the atlas. Here, the right parietal lobe has been turned off, making it easier for the researcher to interactively explore the specific areas of interest. In (D) a slider controls the opacity of all selected connectivities; in (E), users can turn edge bundling on or off for selected brain regions; and in (F), users can set the minimum or maximum threshold values and number of hops to determine which edges to display. Users can change the glyph size of individual nodes or selected brain regions in order to highlight relevant information. Here, the user has selected and enlarged a node (G) and is investigating edges emanating from the left parietal lobe above a threshold of 1.35518. NeuroCave is a web application that runs in both desktop and mobile environments, and users can switch seamlessly between the standard and VR modes on-demand. The inset image (bottom center) shows a neuroscientist (author ADL) exploring the 3D dataset in virtual reality using Oculus Rift VR with Touch controllers.

We introduce NeuroCave, a visual analytics tool to investigate the connectome (Figure 1). Although NeuroCave facilitates a range of explorations, it was initially developed to support clinical neuroscience investigations, primarily related the following analysis tasks:

T1  Identify regions responsible for specific cognitive functions and study their interactions with other regions.
T2  Compare individual networks to the mean or group average connectome, or compare differences between two group average connectomes.

NeuroCave enables researchers to load in different representations of a dataset in order to examine a specific region and to reason about its relationship to other brain regions. We explore an example of this in Use Case 1, in which NeuroCave is used to identify a strong functional coupling between the left and right hemispheres mediated by callosal connections during the resting state. In group studies, individual variations as well as joint network characteristics are studied in order to identify commonalities or differences between groups, including how these change over time. Use Case 2 explores neurological gender differences in connectome datasets and observes how these differences relate to various psychological studies.

NeuroCave enables a wide range of interactive methods to support these tasks, including flexible data loading and data transformations, enabling comparisons within and between connectome datasets; user defined coloring scheme (based on lobar information, modular and/or community affiliation, etc.); a coordinate system that can be defined interactively by users or automatically determined via a modular layout based on platonic solids; adjustable glyph size and transparency of nodes and edges; adjustable connectivity threshold for displaying edges; shortest path between two nodes; on-demand edge bundling and edge coloring; and on-demand labeling of nodes and edges. Details about these interactive functionalities are provided below (in the Methods section), and two use cases present examples of the range of insights that can be generated when using these techniques in NeuroCave to support tasks T1 and T2 (in the Results section).

### Related Work

Many tools exist to generate and visualize the connectome in 2D and 3D (Margulies, Böttger, Watanabe, & Gorgolewski, 2013). Three-dimensional visualization tools most often represent the connectome as node-link diagrams, in which nodes are positioned relative to their corresponding anatomical locations, and links represent the connectivity between nodes. Examples of such tools include the Connectome Visualization Utility (LaPlante, Douw, Tang, & Stufflebeam, 2014), BrainNet Viewer (Xia, Wang, & He, 2013), and the Connectome Viewer Toolkit (Gerhard et al., 2011). In general, node-link diagrams provide an effective overview of the entire graph, which makes it easy to observe relationships between both directly and indirectly connected nodes. However, excessive visual clutter is introduced as the number of edge crossings increases, affecting the readability of the graph.

**Figure 2.**
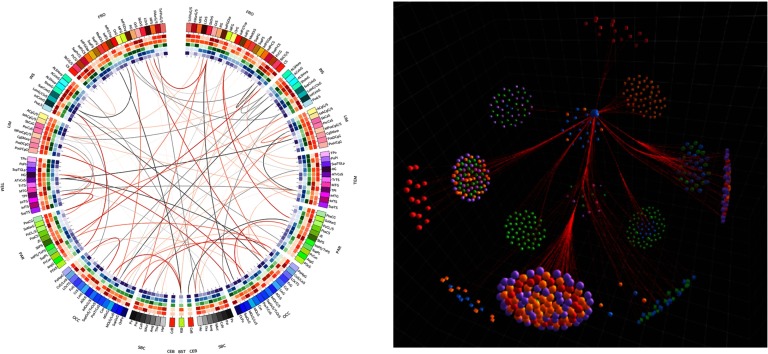
An example of a 2D connectogram (left), taken from the Circos tutorial website (http://circos.ca/tutorials/), versus a 3D platonic solid representation of a connectome and its modularity using NeuroCave (right). With NeuroCave, users can interactively select particular nodes or groups of nodes to explore connectivity on-demand, and alternative layouts based on clustering parameters can be generated as required for a particular analysis task.

Representations of the connectome in 2D are also common. In certain cases, adjacency matrices can better manage large connectome datasets than node-link diagrams (Alper, Bach, Henry Riche, Isenberg, & Fekete, 2013; Ma et al., 2015). However, some visual analysis tasks are difficult to perform using matrix representations (Ghoniem, Fekete, & Castagliola, 2005; Keller, Eckert, & Clarkson, 2006), such as detecting graph alterations in group studies. A popular 2D technique to highlight relevant brain connectivity patterns is the connectogram (Irimia et al., 2012). In a connectogram, the names of each brain region are presented along the perimeter of a circle, and the regions are positioned in two different halves according to the hemisphere they belong to. Furthermore, each hemisphere is broken down into different lobes, subcortical structures, and the cerebellum. The inner space of the circle is divided into multiple-colored nested rings, where each ring shows a heat map representing a specific metric. Interconnections between the regions are illustrated inside the circle by means of curved lines. As with NeuroCave, a goal of the connectogram is to more effectively represent densely connected networks, as is the case for the human connectome. Cacciola et al. (2017) demonstrate that the intrinsic geometry of a structural brain connectome relates to its brain anatomy, noting that the hyperbolic disk seems a congruous space of representation for structural connectomes, one in which it is possible to design brain latent-geometry-based markers for differential connectomic analysis of healthy and diseased. Although connectograms help to prevent some of the clutter that occurs when visualizing networks containing a large number of edges, it can be challenging to correlate anatomical structures with connectivity, and users may find it difficult to make sense of connectograms with many layers of inner and outer circles (Burch & Weiskopf, 2014). Moreover, it can be time-consuming to produce a connectogram by using the popular Circos software (Krzywinski et al., 2009), which requires the preparation of nine distinct configuration files. Finally, lacking a graphical user interface, it is generally used as a presentation tool rather than as a means to interact with connectome data. Although NeuroCave focuses on supporting the analysis tasks defined above, it can be used to represent data in an analogous way. Figure 2 shows an example of similarly dense datasets represented in 2D using a connectogram and in 3D by using NeuroCave.

Although most commonly used visualization tools are dedicated desktop applications, web-based implementations, such as Slice:Drop (Haehn, 2013) or BrainBrowser (Sherif, Kassis, Rousseau, Adalat, & Evans, 2015), free the user from being attached to a specific operating system (Pieloth, Pizarro, Knosche, Maess, & Fuchs, 2013). To this end, NeuroCave is a web-based application and runs in any modern browser, both on desktop and mobile computers. Rojas et al. (2014) finds that the use of stereoscopic techniques can provide a more immersive way to explore brain imaging data, and Hänel, Pieperhoff, Hentschel, Amunts, & Kuhlen (2014) show that healthcare professionals perceive the increased dimensionality provided by stereoscopy as beneficial for understanding depth in the displayed scenery. Moreover, Ware & Mitchell (2008) find that the use of stereographic visualizations reduces the error rate in graph perception for large graphs with more than 500 nodes. Alper, Hollerer, Kuchera-Morin, & Forbes (2011) observe that when coupled with a highlighting technique, stereoscopic representations of 3D graphs outperform their nonimmersive counterpart. NeuroCave harnesses the visualization capabilities of virtual reality (VR) environments, which can facilitate spatial manipulation, identification, and classification of objects and imagery, and aid users in understanding complex scenes (Bohil, Alicea, & Biocca, 2011; Forbes, Villegas, Almryde, & Plante, 2014; Marai, Forbes, & Johnson, 2016). Other tools that make use of VR for visualizing connectomes include AlloBrain (Thompson et al., 2009), BrainX3 (Arsiwalla et al., 2015; Betella et al., 2014), and BRAINtrinsic (Conte et al., 2016; Conte, Ye, Forbes, Ajilore, & Leow, 2015). Similar to BRAINtrinsic, NeuroCave emphasizes the ability to switch between anatomical representations and low-dimensional embeddings of connectome datasets. Although NeuroCave includes some of the virtual reality functionality available in these previous connectome visualization tools, it also enables users to move seamlessly between desktop and VR environments for interactively exploring 3D connectomes in a range of topological spaces, supports larger connectome datasets, includes novel layout strategies for presenting clusters of data in 3D space, and introduces a hardware-accelerated edge bundling technique for reducing link clutter.

Table 1 provides an overview of popular tools used for visualizing connectome datasets. Although each of the visualization software tools listed in Table 1 may partially address the visualization tasks delineated in the introduction, none provides a visualization that can directly facilitate tasks involving various types of comparison between datasets, since they all lack the ability to simultaneously load and synchronize a comparative visualization of multiple connectomes. Instead, the user needs to open multiple instances of the application, which usually requires the use of multiple monitors in order to visually compare the structural or functional connectomes of the same subject, or two subjects belonging to different groups. Clearly, with two instances of the software running, user actions will not be synchronized, making it more difficult to assess visual differences. Some of the applications implemented in scripting languages, such as R and MATLAB, do provide the user with the flexibility to customize views (e.g., to present multiple connectomes simultaneously). However, this requires additional efforts as well as programming expertise. By introducing a side-by-side layout, NeuroCave enables neuroscientists and researchers to efficiently execute tasks that involve comparative analyses, and to simultaneously spot changes occurring within and across subjects. NeuroCave does not target tractography-related usages, which, although an important area of connectomics visualization, are not usually a requirement for clinical neuroscientists (who are the intended audience for our visualization software).

**Table 1.**
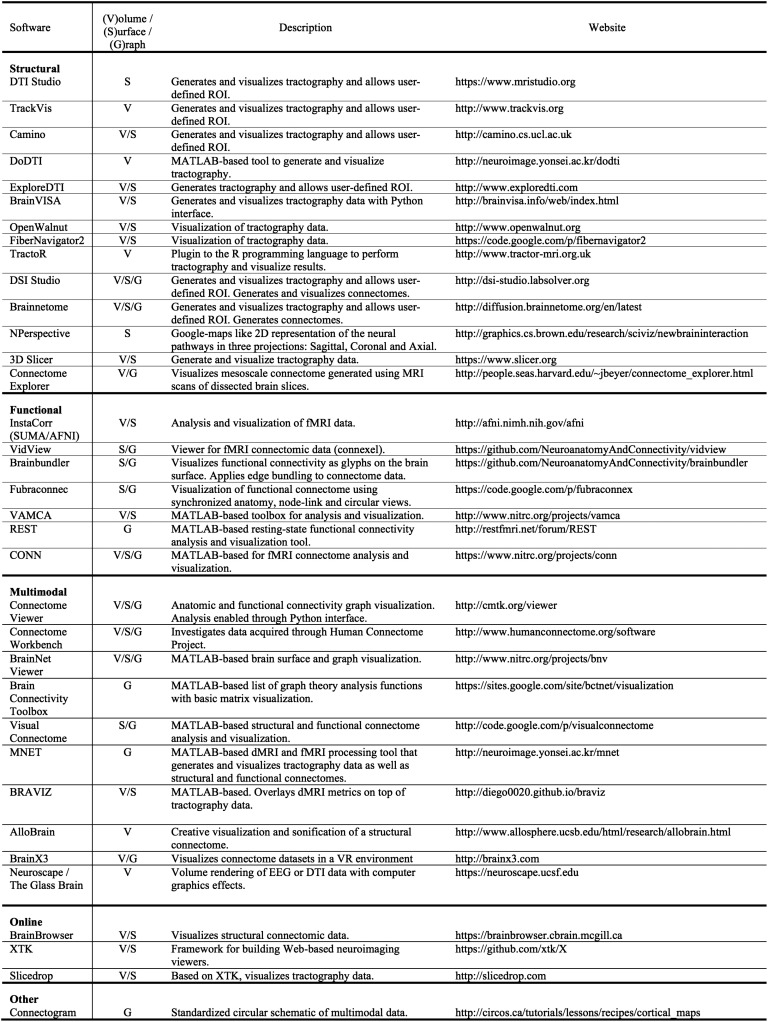
A survey of neuroimaging connectomic software. This table categorizes each software in terms of whether or not it supports structural or functional connectomes, or both, or if the software is accessed online via a browser. Additionally, we indicate whether or not the software visualizes connectomes as a volume, a surface, or as a graph.

## METHODS

**Figure 3.**
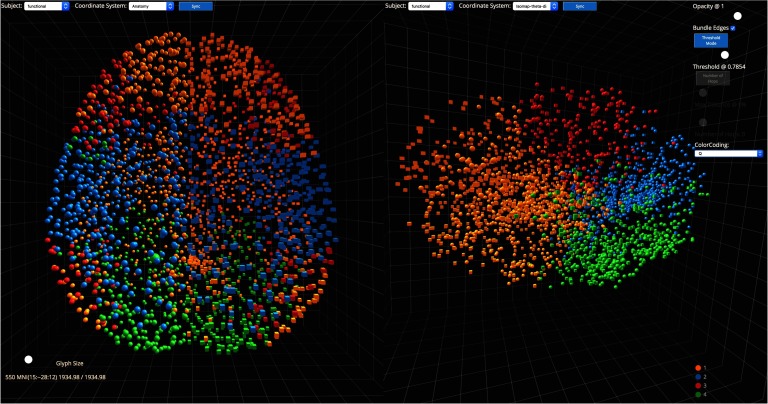
Anatomical versus intrinsic geometry, as first proposed by Ye et al. (2015). Here, we see a screen capture of NeuroCave showing a high-resolution 2514-ROI functional connectome in the anatomical space (left) and the intrinsic space (right), in which nodes that make up a module or “community,” as determined via Q-maximization, clearly form a cluster. Our tool facilitates the simultaneous exploration of multiple connectome datasets in a variety of configurations, enabling researchers to make meaningful comparisons between them and to reason about their differences.

NeuroCave is implemented as a web-based application that makes use of three.js, a JavaScript graphics library for real-time rendering of 3D scenes. It runs on all major web browsers, and is thus platform independent. The default view is formed of two side-by-side rendering views (see Figure 1 and Figure 3). Each view enables the interactive visualization of a connectome as a node-link diagram.

### Group Visualization

NeuroCave loads connectome data from a user-specified folder. This folder must contain all adjacency matrices as well as the corresponding topological and clustering information of the subjects within the study. An index file states the subject ID and its corresponding data files. Each study or analysis session requires a predefined Atlas that provides numerical labels and their corresponding anatomical names to each node. NeuroCave currently supports three Atlases by default: FSL-based parcellation, which consists of 82 labels from FreeSurfer (Fischl, 2012); the Brain Hierarchical Atlas (BHA), comprised of 2,514 labels (Diez et al., 2015); and the Harvard-Oxford Atlas, which uses 177 labels (Makris et al., 1999). Additional Atlases can be created and existing ones can be customized easily, simply by using a preexisting Atlas as a template for defining a new one. (See the online instruction manual available at https://github.com/CreativeCodingLab/NeuroCave for more details of how to load in datasets and customize Atlases; Keiriz, Zhan, Ajilore, Leow, & Forbes, 2018a.)

A common task in disease studies involves the comparison of two groups of subjects—for example, a healthy control group versus a disease group—in order to derive conclusions about alterations due to the disease. Currently, no existing connectome visualization application effectively facilitates real-time simultaneous comparison for two or more datasets. NeuroCave enables neuroscientists to visualize connectome datasets via a synchronized “side-by-side” layout, making it easier to explore differences between groups of subjects, or the same group represented using different spaces, modalities, or in different coordinate systems.

### Topology Visualization

NeuroCave positions nodes according to the provided topological information. Available topologies include the anatomical positioning or any of number of applied transformations that reformulate this positioning into an abstract space. These topologies are automatically identified by the application, and ongoing development aims to enable the transformation of anatomical datasets into a range of topological spaces on-demand. Currently, we have applied a range of dimensionality reduction techniques to connectome datasets, including Isomap (Tenenbaum, De Silva, & Langford, 2000) and t-SNE (Maaten & Hinton, 2008), and we make use of these methods to help identify patterns in the “intrinsic geometry” (i.e., the geometry as determined by the brain connectivity itself, either structural or functional) of a connectome dataset (Cacciola et al., 2017; Ye, Ajilore, et al., 2015). Once these intrinsic datasets are loaded, users can switch between anatomical and these abstract topological spaces as needed to support particular analyses, making it possible to see the same data transformed in various ways in order to investigate the connectome from a range of different perspectives. Figure 3 shows a comparison of the same connectome dataset in an anatomical versus a topological space.

### Clustering Visualization

**Figure 4.**
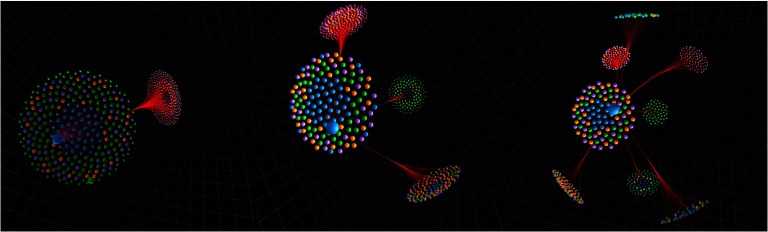
Modular representation on our platonic solid layout can be used to effectively visualize multilevel hierarchical clustering. In this example, each level is a bifurcation of the previous one: level 1 with two clusters (left), level 2 with four clusters (middle), and level 3 with eight clusters (right). Additionally, this figure illustrates how our force-directed edge bundlin (FDEB) algorithm can be used to simplify the visualization of edges; here a node corresponding to the left hippocampus is selected and all emerging edges connected to other brain regions are plotted.

NeuroCave also supports the visualization of clusters of nodes (i.e., modular or community structure), either embedded within a topological space, or simply as groups of related points (where the spatial positioning of a cluster of nodes within the cluster has no meaning). Clustering information is input as a vector of integer values, where each value represents a different module or cluster. When there is no meaningful spatial positioning provided for clusters (or when we choose to exclude this information), NeuroCave makes use of a layout technique that exploits the geometrical properties of platonic solids. In brief, a platonic solid is a regular, convex polyhedron constructed by congruent regular polygonal faces with the same number of faces meeting at each vertex. Five platonic solids exist: tetrahedron, cube, octahedron, dodecahedron and icosahedron, with 4, 6, 8, 12, and 20 faces, respectively. Based on how many clusters are generated, assuming there are less than 20, a suitable platonic solid is chosen such that its number of faces is greater than the number of these clusters. The glyphs of each cluster are then equally distributed according to the sunflower algorithm (Vogel, 1979), covering the corresponding face of a platonic solid embedded in a sphere. This enables the user to “enter” into the geometry (i.e., into the “NeuroCave”) via one of the unpopulated faces, providing a more immersive experience of the data, especially when the VR mode is activated. Users can interactively rearrange the position of the clusters within the platonic solid in order to more easily see particular clusters, for example, those that are densely interconnected, or that are relevant for a particular analysis session. When applicable, clustering can be recomputed on-demand, with the user specifying the number of clusters, which in turn updates the platonic solid that is generated. Connections between individual nodes within clusters or between clusters can be visualized as well, as described below. Figure 4 shows an example of visualizing multilevel hierarchical clustering using our platonic solids approach. Finding appropriate visualizations that are useful for desktop and VR environments (or both) is an ongoing topic in visualization (Liluashvili et al., 2016; Tang, Liu, Zhang, & Mei, 2016). Our above approach is of course just one of the many ways that connectome datasets could be visualized in 3D, and we plan to introduce additional layouts in the future.

### Node and Edge Visualization

By default, we utilize two different glyphs (spheres and cubes) to differentiate between left and right hemisphere affiliation. Nodes can be colored according to lobar or modular information. Controlling nodal transparency is also possible according to their color scheme and colors can be interactively assigned to different amounts of transparency modes as desired. For example, a brain region that has been assigned a particular color can be toggled on or off (made visible or invisible), or the transparency of one or more regions or clusters can be increased or decreased in order to emphasize or de-emphasize them (see Figure 1). The glyph size of individual nodes or groups of nodes is also interactively adjustable by the user via our interface, both in desktop mode or in VR mode. Text labels identifying the nodes can be displayed for all nodes or for user-selected nodes as desired.

NeuroCave includes a range of features to visualize edges efficiently and effectively. Network visualizations that have an excessive amount of overlapping edges, common in dense node-link diagrams, can introduce unwanted visual clutter, which makes it more difficult to read and interpret the network. We implement two ways to mitigate this problem. First, we provide the option to hide all edges by default (i.e., to show only the nodes), and then enable a user to interactively add edges as desired. In this mode, a user can select any node as a “root” node, causing all connected edges stemming from this node to be displayed. Second, to minimize the clutter occurring from edge crossings, we use the force-directed edge bundling (FDEB) algorithm to group edges going in the same direction (Holten & Van Wijk, 2009).

Standard implementations of edge bundling are too slow for the large numbers of edges that can appear in some connectome datasets visualized in NeuroCave, reducing the frame rate of the application and preventing an effective real-time experience. Therefore, we introduce an enhanced WebGL texture-based implementation, extending previous work by Wu, Yu, & Yu (2015). In our approach, we can increase the maximum number of edges between nodes through the use of multiple GPU textures. Our texture-based implementation can bundle the closest 1,000 edges to the selected node at interactive rates on a desktop computer (see Implementation Details below). Although this is sufficient for the datasets we explored, we also enable users to choose threshold values that limit only connections above or below specified strengths to be computed, both to improve performance in situations where the data contains very dense interconnections, and to assist in analyses focused on particular connectivity weights.

**Figure 5.**
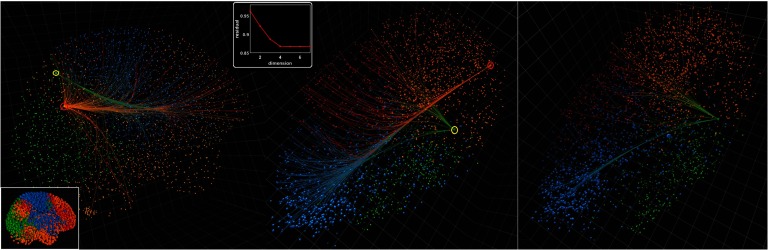
Connectivity emerging from the anterior (red ring) and posterior (yellow ring) parts of the precuneus in anatomical space (left panel) and an “intrinsic” space generated using Isomap (middle panel). Right panel: Connectivity emerging from the posterior part of the precuneus visualized in the intrinsic space. The color code represents the modular structure of the connectome consisting of four modules. Note that the orange community contains the default mode network. The bottom-left inset shows another view of the left panel with all nodes enlarged in order to better see the modular structure. The top-center inset plot shows the residual geodesics for the first 10 dimensions of the Isomap dimensionality reduction algorithm.

Each edge can be colored using a gradient, whose two colors are chosen according to the colors of the source and target nodes that it connects, and where the gradient is skewed toward the node possessing the higher nodal strength. This enables the user to quickly recognize the strength of the selected node with respect to its interconnected neighbors, which can help in identifying important nodes or clusters of nodes, as well as to highlight the reason for modular changes when they occur in group studies. Figure 5 and Figure 7 show examples of this edge coloring approach. (Further details about the layout algorithm, the edge bundling algorithm, and the gradient coloring can be found in the supplementary material available at the project website; Keiriz, Zhan, Ajilore, Leow, & Forbes, 2018a.)

### Virtual Reality

NeuroCave can be viewed on a normal desktop or mobile environment, or via a VR system. Currently we support the Oculus Rift and the Samsung Gear VR platforms, with explicit support for additional platforms planned in the near future. In addition to the standard 3D manipulations of panning, rotating, and zooming, NeuroCave supports the advanced interaction features available on the Oculus Rift via the use of Oculus Rift Touch controllers. The Touch controllers are a pair of VR input devices that track each hand, enabling an effective gesture-based manipulation of the VR environment. The user selects the preview area to be explored in VR and then uses the “thumbsticks” on the Touch devices to navigate the visualized connectome. Nodal selection is enabled via a two-step procedure: first, pressing the grip button lets the user point at and highlight a node; second, pressing the index button selects the highlighted node. We can mimic some of this functionality in other VR platforms (e.g., any platform that supports WebVR, such as Google Cardboard or Daydream), but node selection is not as effortless if the controllers do not contain tracking sensors. Users can enter and leave VR mode as often as they like in order to support investigations of connectome datasets.

From our initial qualitative observations, we find that users are very engaged in exploring the data while in VR mode, and enjoy switching between the different available layouts based on the different clustering and dimensionality reduction techniques. Users also indicate that they appreciate the ability to bring up different datasets on-demand while immersed in the VR mode, especially to see if patterns discovered in one connectome (e.g., a connectome dataset representing average healthy subjects) were present in another (e.g., a connectome dataset representing average diseased subjects). Users also readily move between the desktop display and the VR display without complaint. In practice, users tend to use the VR environments to make initial explorations of the data and to generate hypotheses about the connectomes, and then switch to a desktop view once more nuanced investigations are required. However, we believe this is partly due to users not being as familiar with navigating in VR (and especially with making fine-grained selections with the VR controllers), and also because of the need to use additional applications during an analysis (i.e., for web search, taking notes, etc.) that are not readily available when wearing a portable VR headset.

## RESULTS

NeuroCave has been tested in a wide range of contexts using a variety of datasets. Through using the various features in combination in order to interact with connectome datasets, NeuroCave supports a range of analysis tasks, including identifying relevant brain regions, comparing variations between individual and average connectomes, investigating relationships between structural and functional connectomes, and analyzing group changes in connectomes, among others. The rich set of visualization features provided by NeuroCave makes it possible for users to explore connectome datasets in a flexible manner, to make observations about connectome data, to generate hypotheses about these observations, and then to dive in more deeply to support or invalidate hypotheses. Here, we briefly describe two use cases in which neuroscientists use NeuroCave to analyze connectome datasets. Although these use cases show only preliminary research results, they provide evidence indicating that NeuroCave facilitates useful explorations of complex datasets. That is, NeuroCave supports the process of generating and querying visual representations in order to answer task-specific questions, or what Russell, Stefik, Pirolli, and Card (1993) have described as “sensemaking.”

### Use Case 1

Our first use case explores a resting-state fMRI high-resolution dataset (Keiriz, Zhan, Ajilore, Leow, & Forbes, 2018b) consisting of 2,514 regions-of-interest publicly available at NITRC (http://www.nitrc.org/frs/?group_id=964) and demonstrates the feasibility of visualizing the intrinsic geometry of the resting state. Extending the PACE procedure introduced by Zhan et al. (2017) for an *N* × *N* functional connectome, this intrinsic geometry is reconstructed by first estimating the probability that an edge *e*_*ij*_ is positive (nodes i and j are co-activating) or negative (nodes i and j are anti-activating) using a group of subjects. The resulting probabilities, the edge positivity positivity *EP*_*ij*_ and edge negativity *EN*_*ij*_ form a complementary pair, since *EP*_*ij*_ + *EN*_*ij*_ = 1, and thus can be jointly coded using the angle of a unit-length vector: $\theta_{ij}=\arctan(\sqrt{EN_{ij}/EP_{ij}})$ that varies from 0 to 90 degrees. Using dissimilarity graph embedding (Xing et al., 2016), each node, *i*, is then embedded in a 2,514-dimensional space at the coordinates (*θ*_*i*1_, *θ*_*i*2_, …*θ*_*i*2514_)^*T*^. Here, we picked the classic Isomap algorithm because of its quasi-isometry property (Tenenbaum et al., 2000) that aims to preserve geodesic distances in a lower dimensional space (i.e., the “intrinsic space”), but other type of low-dimension embedding approaches can be used. Separately, we determined the community structure by maximizing the Q-modularity metric, yielding four communities in this case (Newman, 2006).

**Figure 6.**
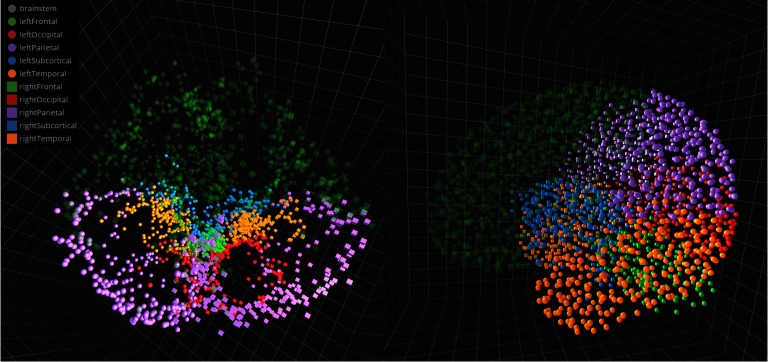
This figure shows an exploration of the structural connectome in intrinsic (left) and anatomical (right) geometry for the same connectome dataset gathered from the same participants as the functional connectome shown in Figure 5.

From the inset plot in Figure 5, it is clear that the Isomap-derived intrinsic geometry of the resting-state functional connectome is achieved with four dimensions, a novel finding that merits further research. To enable 3D visualization of the transformed topology, we retained the first three dimensions of Isomap and visualized the modular structure of the brain in both the anatomical space as well as this intrinsic space. As the community affiliation is separately determined using Q-maximization, it is thus validating to see that the nodes assigned to the same community according to Q are positioned close to one another in the intrinsic space. To illustrate how neuroscientists explore this complex topological space and gain further insight into the brain, we selected two nodes that belong to the anterior and posterior part of the precuneus. Although the nodes are anatomically close to each other, they are known to be functionally distinct (and thus belong to different modules). Indeed, the anterior part of the precuneus is an important region of the default mode network known to be responsible for self-referential imagery (thinking about self) and is involved in autobiographical tasks and self-consciousness, thus activated during “resting consciousness” (Cavanna, 2007). As Figure 5 illustrates, in this intrinsic space the anterior part of the precuneus, while assigned to the orange module that contains the default mode network, also exhibits diverse connections with various regions of the brain: the blue cluster that contains the sensorimotor module and the red module that contains the frontoparietal executive or task-positive system. By contrast, the posterior precuneus is part of the visual system (green) and has a relatively restricted pattern (compared with its more anterior counterpart) of connectivity with the rest of the brain. Notably, such connectivity differences only become visually apparent when visualized in this novel space. The intrinsic geometry of the mean structural connectome from the same dataset reveals a completely different topology, shown in Figure 6, left. Here, note that visually there exists a strong left-right symmetry, which is not present in the case of functional connectome. Put together, these two intrinsic geometries suggest strong functional coupling between the left and right hemispheres, likely mediated by the callosal connections, during the resting state. This finding is consistent with recent results reported by Cacciola et al. (2017), who indicate a clear matching between intrinsic geometry and neuroanatomy.

This use case highlights our visualization system successfully supporting task T1, enabling neuroscientists to explore high-density connectomics data comprising a large number of regions of interest (ROIs) in order to identify and further understand the specific functionality of different brain regions. Moreover, the side-by-side visualization enables users to reason about the relationships between the anatomical and the intrinsic topology, facilitating further insight into how the same brain region can take part in different tasks.

### Use Case 2

**Figure 7.**
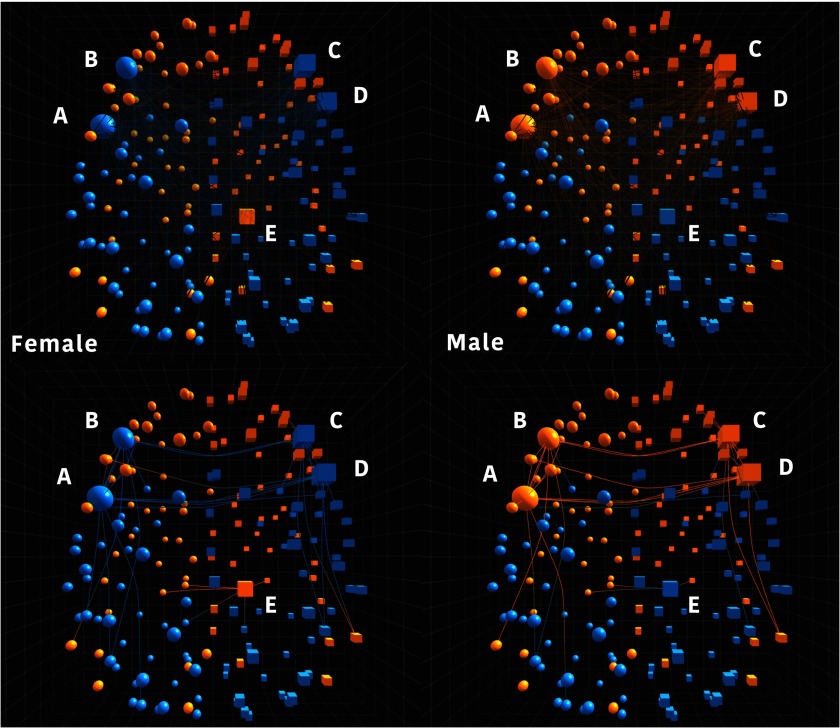
This figure shows two annotated screen captures taken during a visual analysis session using NeuroCave in which a researcher interactively explores sex-specific resting-state functional connectomes in the F1000 repository, exploring differences between average female (left) and male (right) connectomes. Here, the color code represents the hierarchical modularity of the connectome, represented as a dendrogram at the most global level (2 modules). All edges are turned on and nodes that exhibited switching in modularity are selected (indicated with the larger marker size). Node A is from the left precentral gyrus, B and C are nodes from the bilateral most posterior segment of the frontal pole near the fronto-temporal junction, D is from the right inferior frontal gyrus pars opercularis, and E is from the right hippocampus. Contrasting the top image with the bottom image (where edges were thresholded at a higher value of 0.33), sex-specific patterns reveal themselves, showing a tightly interconnected cluster in A–D, whereas E exhibits an opposite switching pattern.

Our second use case investigates the sex-specific resting-state functional connectomes in the F1000 repository, a large 986 subject publicly available resting-state fMRI connectome dataset available at https://www.nitrc.org/projects/fcon_1000/. Recognizing that negative edges between nodes in connectome datasets may be neurobiologically relevant (Sporns & Betzel, 2016), Zhan et al. (2017) introduce an approach that utilizes negative signal correlations between two nodes. The following postprocessing steps were performed on the F1000 dataset: (a) First, to eliminate the potential confounding effect of age, we only included subjects between 20 to 30 years old (319 women at 23.25 ± 2.26 years of age and 233 men at 23.19 ± 2.35); and (b) using PACE (probability-associated community estimation), we constructed a hierarchical modularity of the resting-state functional connectome followed by a rigorous permutation testing procedure that established novel sex differences, statistically significant across all levels of modular hierarchy, starting at the first level (*p* = 0.0378), for the following regions, as indicated in Figure 7: left precentral gyrus (A), bilateral most posterior segment of the frontal pole near the frontotemporal junction (B and C), right inferior frontal gyrus pars opercularis (D), and right hippocampus (E). These observations, which were initially noted in explorations using NeuroCave, were then further analyzed using other statistical tools, as discussed in Zhan et al. (2017).

NeuroCave enables us to visually interpret these sex differences as follows. First, we examine the connectivity patterns of the aforementioned regions in the anatomical (first hierarchical level) space by selecting them in NeuroCave (Figure 7, top) and using the threshold sliders available in NeuroCave to restrict edges to values with absolute correlation values for fMRI BOLD signals larger than 0.33 (Figure 7, bottom). In the bottom image of Figure 7, it is clear that A–D forms a tightly interconnected cluster, whereas E (the right hippocampus) exhibits an opposite switching pattern. In fact, affiliation patterns for the right hippocampus differ between sexes. In women they are clustered with other regions that collectively form the default mode network, or DMN, the orange module, whereas in males they are clustered with other parietal and occipital non-DMN ROIs. Notably, the affiliation differences are in the opposite direction for the left and right frontal poles, the left precentral gyrus, and the right pars opercularis, such that they are assigned the blue module (i.e., non-DMN) in the average female connectome.

The left and right frontotemporal junctions form the larger language system, with the right pars opercularis (functionally coupled with its homologous area on the left that forms the Broca’s language area) linked to the processing of semantic information (Heim et al., 2005), and the superior temporal gyrus involved in the comprehension of language, as well as in the perception of emotions in facial stimuli (Bigler et al., 2007). Thus, the observed connectivity differences are likely related to well-known sex differences in language and emotion/affect processing, as well as differences in self-referential/autobiographical information retrieval. By contrast, the hippocampus is known to play an important role in the formation of new memory, retrieval of declarative long-term memory, and the management and processing of spatial and spatiotemporal working memory. The modular affinity between the right hippocampus and other non-DMN regions in the parietal and occipital lobes in men may thus be related to their postulated advantage in visuospatial tasks, including spatial visualization, perception, and mental rotation (Linn & Petersen, 1985). (The visual system is heavily composed of the occipital lobe, which is responsible for first-level visual processing, although part of the parietal lobe is instrumental for visuospatial skills.)

Additionally, examining the same dataset in NeuroCave, we find a clear visually indication that the right hippocampus exhibits a stronger contralateral connectivity with the left frontal cortex in women than in men. Interestingly, in ovariectomized female rates there is a decrease in dendritic spine in the prefrontal cortex (PFC) and hippocampus (Wallace, Luine, Arellanos, & Frankfurt, 2006), whereas in human studies estrogen has been shown to activate the same regions (Berman et al., 1997; Maki, 2005; Maki & Resnick, 2000; Stevens, Clark, & Prestwood, 2005) and estrogen infusion in a group of post-menopausal women increased the connectivity between the two (Ottowitz et al., 2008).

The discovery of such subtle differences is dependent on an iteratively explorative visualization process, only possible with the comprehensive suite of tools, such as those we have implemented in NeuroCave. Indeed, NeuroCave provided researchers with the ability to visually explore the sex-specific resting-state functional connectomes, leading to initial hypotheses, some of which have been analyzed much more thoroughly in Zhan et al. (2017). This use case demonstrates the effectiveness of NeuroCave in supporting task T2, enabling neuroscientists to better understand neurological differences between connectome datasets and to observe how these differences relate to various psychological studies.

## DISCUSSION

As demonstrated by these two use cases, NeuroCave enables brain researchers to conduct sophisticated explorations of large datasets interactively. It provides users with the ability to navigate connectomes in a flexible manner, using the available interactive techniques to switch between different views, to change clustering metrics and layouts, to highlight meaningful connections and to filter out edges or nodes not relevant to a specific inquiry, to see data in different topological spaces, and to move seamlessly in and out of VR mode. In both use cases described above, researchers made use of each of these techniques in order to home in on interesting features of the connectome data, and to then validate initial hypotheses about these features.

### Implementation Details

Within NeuroCave, node selection and the edge display are synchronized between the two side-by-side views: selecting a node in one view activates the node and displays its corresponding edges across both viewing areas, independent of the chosen configuration for each side. In order to make real-time manipulation of large datasets possible, we use hardware-accelerated graphics and extend a texture-based implementation of the FDEB algorithm (Wu et al., 2015). Our implementation harnesses the computational power of the graphical processing unit (GPU) in order to perform the required computations, and is at least 50 times faster than its CPU counterpart, enabling real-time edge bundling of over 1,000 edges at interactive rates (tested on a desktop computer with the following hardware: Intel Core i7, 3.4 GHz CPU, a Nvidia GTX 1070 GPU card, and 32GB RAM). The other features presented in NeuroCave do not require a dedicated GPU, and the real-time edge bundling does not need to be active to use the software. However, systems with a dedicated GPU are able to render much larger networks without any noticeable lag between frames. The datasets investigated in Use Case 1, where each panel displays 2,514 nodes, are the largest that we have so far tested, and our system successfully facilitates pair-wise comparisons between two groups or the same group presented in different topological spaces. Although only two panels are displayed simultaneously, a user can swap out the dataset for each panel on-demand. The ability to do this is limited only by the memory available to the browser. In practice, we have loaded over a dozen datasets into NeuroCave at the same time, with no loss in performance.

### Conclusion

In this paper we presented NeuroCave, a web-based, VR-compatible visualization system that enables researchers to explore the human connectome in a range of immersive, interactive environments, as well as within traditional desktop or mobile environments. NeuroCave supports the comparison of two datasets in a side-by-side layout in order to facilitate the discovery of connectivity (or disconnectivity) patterns in group studies. Our software makes use of the GPU to greatly improve the rendering speed of large connectome datasets and to enable real-time user interactions. As shown in the two illustrative cases, we believe that NeuroCave is a valuable tool for a wide range of structural and functional connectome analyses. Future work will give users the option to make use of popular community detection algorithms, such as Louvain (Blondel, Guillaume, Lambiotte, & Lefebvre, 2011) and Infomap (Rosvall & Bergstrom, 2008), which were found to perform well across a range of benchmarks (Lancichinetti & Fortunato, 2009). We also will adapt our system to support temporally varying dynamic datasets (Crossley et al., 2016; Forbes et al., 2018; Ma, Forbes, Llano, Berger-Wolf, & Kenyon, 2016; Purgato, Santambrogio, Berger-Wolf, & Forbes, 2017), and investigate how NeuroCave can facilitate comparisons between structural and functional connectomes in order to reveal the complex mappings between them (Bullmore & Sporns, 2009; C. Honey et al., 2009; C. J. Honey, Kötter, Breakspear, & Sporns, 2007). Another future goal is to quantitatively assess the impact of VR mode on analysis tasks and empirically investigate the current NeuroCave workflow, which encourages moving between VR and desktop modes. Additionally, we will investigate the use of collaborative VR environments in which multiple users can interact with the same datasets simultaneously (Marrinan et al., 2016, 2017).

## SUPPORTING INFORMATION

The NeuroCave application and open source code, along with detailed instructions and examples, are freely available for use and for download at our GitHub repository: https://github.com/CreativeCodingLab/NeuroCave. All figures in this article were made using NeuroCave, and the datasets supporting the use cases can be explored via the online version of NeuroCave at https://creativecodinglab.github.io/NeuroCave/.

## AUTHOR CONTRIBUTIONS

Johnson Keiriz: Software; Visualization; Writing – original draft; Writing – review & editing. Liang Zhan: Data curation; Validation. Olusola Ajilore: Conceptualization; Investigation; Methodology; Project administration; Resources; Supervision; Validation; Writing – review & editing. Alex Leow: Conceptualization; Formal analysis; Funding acquisition; Investigation; Methodology; Project administration; Resources; Supervision; Validation; Writing – original draft; Writing – review & editing. Angus G. Forbes: Conceptualization; Investigation; Methodology; Project administration; Resources; Software; Supervision; Validation; Visualization; Writing – original draft; Writing – review & editing.

## FUNDING INFORMATION

Alex Leow, National Institutes of Health (http://dx.doi.org/10.13039/100000002), Award ID: R21AG056782.

## TECHNICAL TERMS

Edge bundling: techniques that reduce visual clutter in a network by grouping together edges that follow similar trajectories between nodes or clusters of nodes.
Visual clutter: the state in which the representation of data leads to a degeneration of performance at some visual analysis task. For example, networks with many edge crossing introduce visual clutter because it becomes more difficult to identify paths between two nodes.
Intrinsic geometry: the very high dimensional nonlinear geometric space natively induced by the brain’s interconnectivity as encoded by the connectome, either structural or functional, instead of the neuroanatomical space.
Sensemaking: the process of building a mental model by exploring and organizing data, facilitating decision making and problem solving.
Q-modularity: a scalar metric that measures a network’s community or modular structure. It is computed as the fraction of edges that fall within the given modules minus the expected fraction if edges were randomly distributed. Dense nodal connections within the same modules and sparse connections between nodes in different modules are found in networks with high modularity.
Default mode network, or DMN: a network that comprises different brain regions jointly active during the resting state.
Probability-associated community estimation, or PACE: a noncorrelation-based computational connectomics approach to the modularity of the functional connectome, in which co- versus antiactivations naturally occur between brain regions.
